# Fatal outcome in isolated Pauci‐immune pulmonary capillaritis: A case report

**DOI:** 10.1002/rcr2.70051

**Published:** 2024-10-17

**Authors:** Zeinab El Mawla, Ghinwa Hammoud, Racha Abed El Hamid, Abbas Zreik, Ali Tfayli, Bassam Mansour

**Affiliations:** ^1^ Department of Pulmonary & Critical Care, Faculty of Medical Sciences Lebanese University Hadat Lebanon; ^2^ Department of Internal Medicine, Faculty of Medical Sciences Lebanese University Hadat Lebanon; ^3^ Cardiology Division Zahraa Hospital, University Medical Center (ZHUMC) Beirut Lebanon; ^4^ Pulmonary and Critical Care Division Zahraa Hospital, University Medical Center (ZHUMC) Beirut Lebanon

**Keywords:** case report, diffuse alveolar haemorrhage, hemoptysis, isolated Pauci‐immune pulmonary capillaritis, pericardial effusion, pulmonary vasculitis

## Abstract

Isolated Pauci‐immune pulmonary capillaritis (IPIPC) is a rare form of small vessel vasculitis that affects only the lungs, causing inflammation of pulmonary capillaries and potentially leading to severe outcomes like alveolar haemorrhage. A 23‐year‐old woman with a prior diagnosis of rheumatoid arthritis presented with hemoptysis and respiratory distress, ultimately diagnosed with IPIPC. Despite treatment with high‐dose steroids and intravenous immunoglobulin, her condition deteriorated, resulting in respiratory failure and death. IPIPC often lacks systemic symptoms and ANCA positivity, complicating diagnosis and treatment. Imaging, bronchoscopy, and histopathology are key for diagnosis, while management typically involves corticosteroids and possibly immunosuppressives. The case underscores the challenges in identifying and treating IPIPC, highlighting the importance of early intervention to improve prognosis, even though complications can still lead to significant respiratory issues and mortality.

## Graphical abstract

In this report, we present a rare case of isolated Pauci‐immune pulmonary capillaritis (IPIPC) that proved refractory to conventional medical therapies.

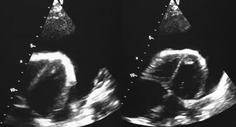

## INTRODUCTION

Vasculitis encompasses a group of disorders characterized by cytokine‐mediated alterations in endothelial cells, resulting in aberrant leukocyte activation and subsequent damage to blood vessels of varying sizes.^1^ Pulmonary vasculitis specifically refers to the inflammation of small blood vessels within the lungs, leading to substantial destruction and damage of lung parenchyma. This condition can present with severe complications, including diffuse alveolar haemorrhage and respiratory failure.^2^, ^3^

A particularly rare and challenging subtype of pulmonary vasculitis is Isolated Pauci‐Immune Pulmonary Capillaritis (IPIPC). IPIPC is distinguished by its presentation of pulmonary manifestations, such as alveolar haemorrhage, occurring in the absence of systemic disease and with negative serological markers. This form of vasculitis is notable for its isolated pulmonary involvement, which can complicate diagnosis and management due to the lack of systemic features.^4^, ^5^

Advancements in therapeutic strategies over recent years have significantly impacted the management of pulmonary vasculitis. Treatments including corticosteroids, cyclophosphamide, intravenous immunoglobulin (IVIG), and rituximab have been associated with reduced mortality rates and increased rates of disease remission. However, despite these advancements, the management of IPIPC remains complex and can be particularly challenging.^6^

In this report, we present a rare case of IPIPC that proved refractory to conventional medical therapies.

## CASE REPORT

A 23‐year‐old female, non‐smoker, presented with a 2‐week history of shortness of breath, chest discomfort, and generalized fatigue. The patient also reported mild abdominal swelling and a decreased ability to perform activities of daily living. She had been healthy prior to this episode and was previously pregnant, with her delivery occurring 9 months prior to presentation.

The patient denied any accompanying fever, respiratory symptoms, gastrointestinal or genitourinary issues. A thorough review of systems was negative for weight loss, arthralgia, rash, or recurrent miscarriages. At the time of presentation, the patient was tachycardic with a heart rate of 115 beats per minute. However, her oxygen saturation was normal at 97% on room air, and her blood pressure was 126/76 mmHg.

**FIGURE 1:**
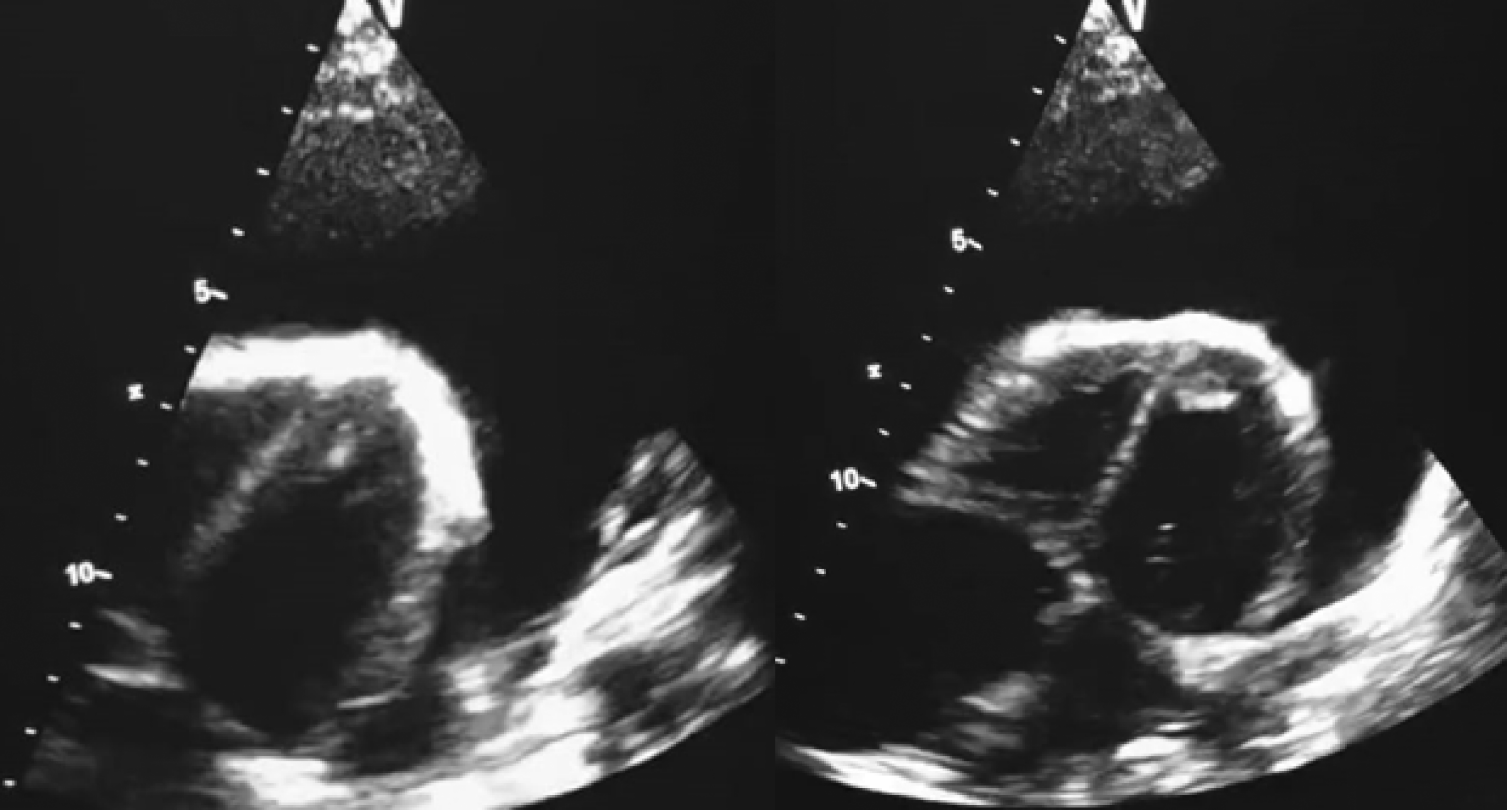
An echocardiogram demonstrates a severe circumferential pericardial effusion.

A computed tomography (CT) scan of the chest revealed a severe pericardial effusion with a maximal thickness of 38 mm. An urgent echocardiogram demonstrated a normal‐sized and functioning left ventricle (LVEF 60%), no significant valvulopathy, but severe circumferential pericardial effusion causing right ventricular compression and inferior vena cava (IVC) plethora, indicative of acute cardiac tamponade (Figure 1).

A pericardial‐peritoneal window was performed, resulting in the drainage of 900 cc of hemorrhagic fluid. Microbiological cultures, including bacterial, fungal, and acid‐fast stains, were negative. Thyroid function tests were normal, and cytology of the pericardial fluid did not show malignancy. Pericardial biopsy revealed focal lymphoid follicular hyperplasia, consistent with an inflammatory reaction. Autoimmune serologies showed a slightly positive rheumatoid factor (RF) and Ro‐52 but were otherwise negative (ANCA profile, ANA, anti‐dsDNA, anti‐Scl‐70, and anti‐GBM). Kidney function remained normal, and the patient exhibited no articular or skin manifestations.

Following clinical improvement, the patient was discharged with a diagnosis of rheumatoid arthritis with isolated pericardial involvement and prescribed prednisone, colchicine, and hydroxychloroquine.

**FIGURE 2:**
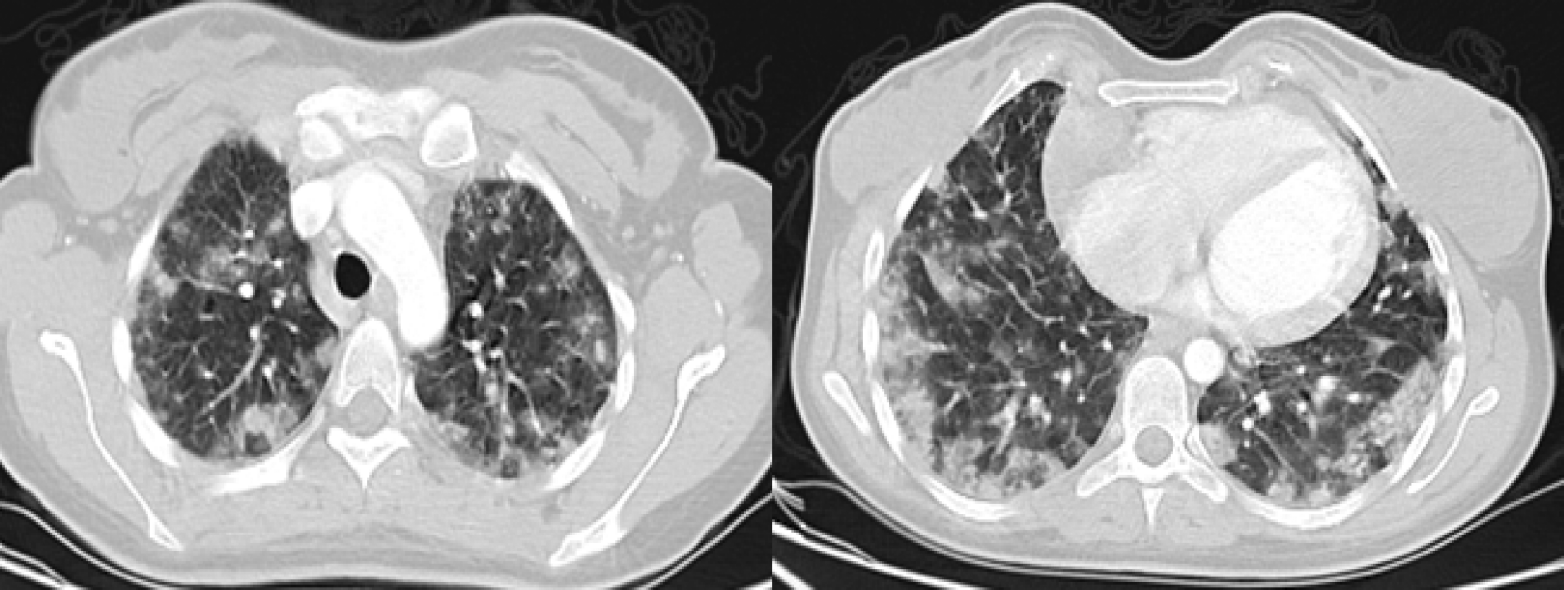
A computed tomography chest scan showed diffuse alveolar ground‐glass infiltrates predominantly in the peripheral lung fields, affecting 60%–70% of the bilateral lung parenchyma.

Two months later, the patient was readmitted with hemoptysis and dyspnea. She was hypoxemic and required 5 L/min oxygen by nasal cannula. Laboratory findings showed haemoglobin at 8 g/dL. A CT chest scan demonstrated diffuse alveolar ground‐glass infiltrates predominantly in the peripheral lung fields, affecting 60%–70% of the bilateral lung parenchyma (Figure **2**). Bronchoscopy with transbronchial biopsy revealed thick‐walled vessels with significant inflammation, consistent with partially treated vasculitis. A diagnosis of IPIPC was made.

Treatment with pulse methylprednisolone (500 mg IV daily for 3 days) resulted in dramatic improvement and resolution of symptoms. The patient was discharged on high‐dose prednisone.

**FIGURE 3:**
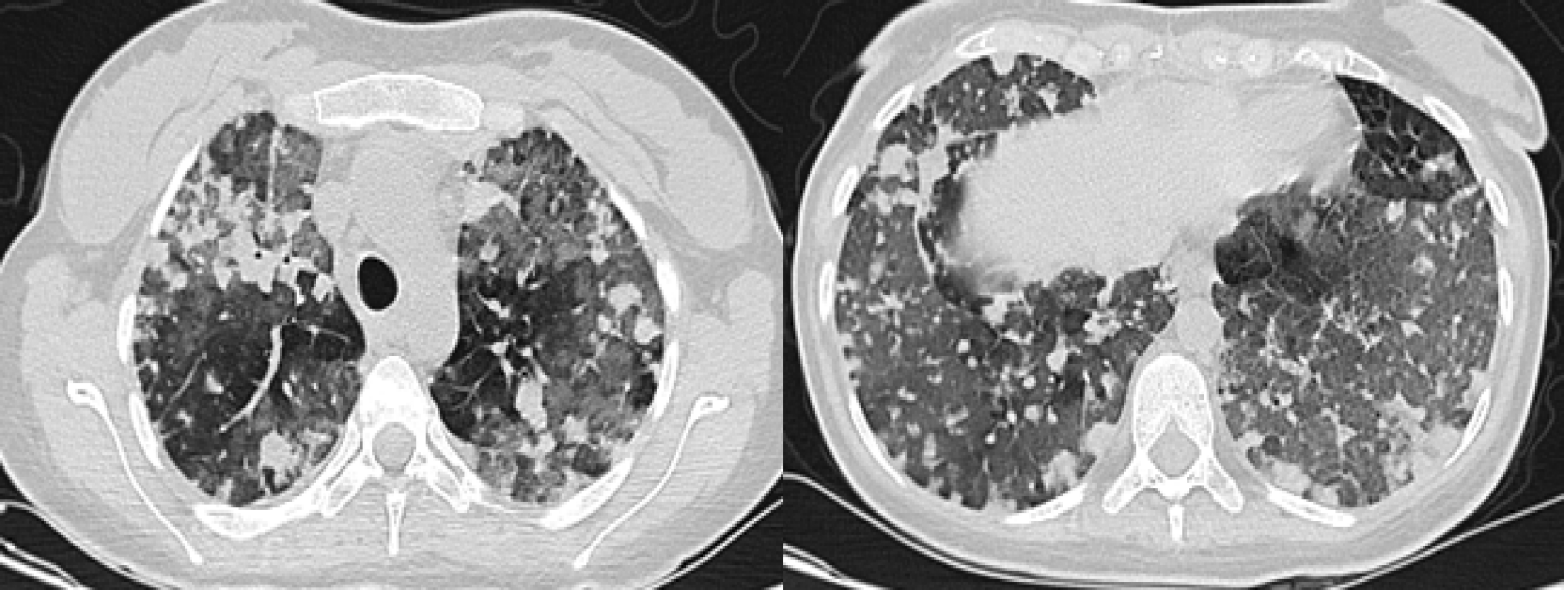
A computed tomography chest scan revealed numerous nodular opacities of varying sizes and extensive alveolar haziness, consistent with diffuse alveolar haemorrhage.

Four days after discharge, the patient re‐presented with massive hemoptysis and severe dyspnea. Laboratory results showed haemoglobin at 6 g/dL. A repeat CT chest scan revealed numerous nodular opacities of varying sizes and extensive alveolar haziness, consistent with diffuse alveolar haemorrhage (Figure 3) Two units of packed red blood cells were transfused. Due to the unavailability of cyclophosphamide and rituximab, pulse steroid therapy with 1 g of methylprednisolone for 3 days was initiated, along with intravenous immunoglobulin (IVIG).

Despite these interventions, the patient's condition rapidly worsened, leading to severe respiratory distress and subsequent intubation. Plasmapheresis was performed for refractory IPC, but the patient did not respond to the treatment and tragically passed away.

## DISCUSSION

IPIPC is a rare form of small vessel vasculitis that predominantly affects the capillaries in the lungs, leading to pulmonary haemorrhage and damage to the alveoli. This condition is characterized by minimal or no systemic involvement typically seen in other small vessel vasculitides. It is distinguished by the lack of classical anti‐neutrophil cytoplasmic antibody (ANCA) positivity that is commonly associated with diseases such as granulomatosis with polyangiitis (GPA) or microscopic polyangiitis (MPA).^4^, ^5^

The epidemiology of IPIPC indicates it is a rare condition with an incidence estimated at 1–2 cases per million annually. Although precise data are scarce due to its rarity and potential for misdiagnosis, it is known to affect adults, with a slight predominance in males and typically presents in middle‐aged individuals.^7^ The rarity of the condition makes it challenging to gather extensive epidemiological data.

The causes of IPIPC remain largely idiopathic. There is no definitive association with known systemic vasculitis markers, and it is not consistently linked to infections, drug reactions, or other environmental factors, though such associations are occasionally noted.^8^, ^9^ The absence of systemic symptoms differentiates it from other systemic vasculitides.

Patients with IPIPC typically present with symptoms such as hemoptysis, which is the most common and distinctive symptom. Other symptoms include dyspnea (shortness of breath) and cough, often associated with respiratory distress. Systemic symptoms such as fever or fatigue are less common, and the absence of joint pain or skin rash helps differentiate this condition from other systemic vasculitides.^10^

Diagnosis is based on a combination of imaging, bronchoscopy, and histopathological findings. Chest CT scans often reveal diffuse alveolar haemorrhage, ground‐glass opacities, and nodular infiltrates that are suggestive but not definitive. Bronchoscopy and bronchoalveolar lavage (BAL) can provide samples that show hemosiderin‐laden macrophages, supporting the diagnosis of alveolar haemorrhage.^11^, ^12^ Histopathology from lung biopsies typically reveals inflammatory changes in the capillary walls with minimal immune complex deposition, distinguishing it from other types of vasculitis.^9^ Autoimmune testing is essential to rule out other forms of vasculitis, even though ANCA testing is usually negative in this condition.^13^

Treatment for IPIPC primarily involves corticosteroids. High‐dose corticosteroids, such as prednisone or methylprednisolone, are commonly used to manage inflammation. In severe or refractory cases, additional immunosuppressive agents like cyclophosphamide or rituximab may be required.^14^, ^15^ Supportive care, including managing complications like anaemia and respiratory support, is also crucial.

Complications of this condition include diffuse alveolar haemorrhage, which can lead to significant respiratory distress and potentially require mechanical ventilation. Chronic inflammation can result in long‐term pulmonary damage, including pulmonary fibrosis.^16^, ^17^ Prompt and effective treatment is essential to manage these complications and improve outcomes.

The prognosis for IPIPC varies. Many patients experience significant improvement and resolution of symptoms with appropriate treatment. However, delayed diagnosis or inadequate management can lead to persistent respiratory issues or progressive lung damage. Early and aggressive treatment typically yields a favourable outcome, but ongoing monitoring is necessary to manage potential relapses or complications.^10^

In conclusion, the case of IPIPC illustrates the diagnostic and therapeutic challenges inherent in managing this rare and severe condition. Despite initial treatment success with corticosteroids, the patient ultimately succumbed to complications, including diffuse alveolar haemorrhage and refractory disease. This highlights the importance of early and accurate diagnosis through imaging, bronchoscopy, and histopathology, as well as the need for aggressive treatment strategies to manage the disease effectively. The case underscores the critical need for heightened awareness and prompt intervention to improve outcomes and prevent severe complications in patients with IPIPC.

## FUNDING INFORMATION

The authors declared that this study has received no financial support.

## CONFLICT OF INTEREST STATEMENT

None declared.

## ETHICS STATEMENT

Written informed consent was obtained from the patient for publication of this case report and any accompanying images. A copy of the written consent is available for review upon request by the Editor‐in‐Chief of this journal.

## Data Availability

The data that support the findings of this study are available on request from the corresponding author. The data are not publicly available due to privacy or ethical restrictions.
